# MicroRNAs and Long Non-Coding RNAs as Potential Candidates to Target Specific Motifs of SARS-CoV-2

**DOI:** 10.3390/ncrna7010014

**Published:** 2021-02-18

**Authors:** Lucia Natarelli, Luca Parca, Tommaso Mazza, Christian Weber, Fabio Virgili, Deborah Fratantonio

**Affiliations:** 1 Institute for Cardiovascular Prevention (IPEK), Ludwig-Maximilians-Universität (LMU), 800336 Munich, Germany; 2 IRCCS Casa sollievo della Sofferenza, Laboratory of Bioinformatics, 71013 San Giovanni Rotondo (FG), Italy; l.parca@css-mendel.it (L.P.); t.mazza@css-mendel.it (T.M.); 3 German Center for Cardiovascular Research (DZHK), Partner Site Munich Heart Alliance, 80336 Munich, Germany; 4 Department of Biochemistry, Cardiovascular Research Institute Maastricht (CARIM), Maastricht University, 6200 MD Maastricht, The Netherlands; 5 Munich Cluster for Systems Neurology (SyNergy), 81377 Munich, Germany; 6 Council for Agricultural Research and Economics, Research Center for Food and Nutrition, 00178 Rome, Italy; fabio.virgili@crea.gov.it; 7 Biotechnology and Biopharmaceutics, Department of Biosciences, University of Bari Aldo Moro, 70125 Bari, Italy; deborah.fratantonio@uniba.it

**Keywords:** oligosequences, SARS-CoV-2, COVID-19, target therapy, non-coding RNAs

## Abstract

The respiratory system is one of the most affected targets of SARS-CoV-2. Various therapies have been utilized to counter viral-induced inflammatory complications, with diverse success rates. Pending the distribution of an effective vaccine to the whole population and the achievement of “herd immunity”, the discovery of novel specific therapies is to be considered a very important objective. Here, we report a computational study demonstrating the existence of target motifs in the SARS-CoV-2 genome suitable for specific binding with endogenous human micro and long non-coding RNAs (miRNAs and lncRNAs, respectively), which can, therefore, be considered a conceptual background for the development of miRNA-based drugs against COVID-19. The SARS-CoV-2 genome contains three motifs in the 5′UTR leader sequence recognized by selective nucleotides within the seed sequence of specific human miRNAs. The seed of 57 microRNAs contained a “GGG” motif that promoted leader sequence-recognition, primarily through offset-6mer sites able to promote microRNAs noncanonical binding to viral RNA. Similarly, lncRNA H19 binds to the 5′UTR of the viral genome and, more specifically, to the transcript of the viral gene Spike, which has a pivotal role in viral infection. Notably, some of the non-coding RNAs identified in our study as candidates for inhibiting SARS-CoV-2 gene expression have already been proposed against diverse viral infections, pulmonary arterial hypertension, and related diseases.

## 1. Introduction

The virus causing COVID-19 disease, severe acute respiratory syndrome coronavirus 2 (SARS-CoV-2), is a member of the coronaviruses family, causing acute respiratory distress syndrome (ARDS), acute lung injury (ALI), and in chronic stages, pulmonary failure and death [1,2]. One of the reasons proposed for the extremely high transmission rate that characterizes SARS-CoV-2 resides in the high mutation rate in the Spike nucleotide sequence [3], making viral infection more rapid. Several therapeutic approaches have been considered so far [4], with a wide range of efficacy, frequently associated with important side effects [5]. Clinically approved, effective vaccines against SARS-CoV-2 have just been made available and data on the first ongoing vaccinations suggest promising beneficial preventions against viral infection. However, effective therapeutic strategies are still needed while waiting for significant “herd immunity” of the whole population [6].

Small interference RNAs (siRNAs) and long RNA antisense locked nucleic acid (LNA) oligos are catching the interest of the scientific community as therapeutic strategies in diverse pathologies, due to low side-effects and high efficiency ratio in vivo [7,8]. Indeed, non-coding RNA (ncRNA)-based drugs can mimic the mechanisms of many endogenous RNAs [9,10,11]. Under this perspective, miRNA-based drugs have already been utilized to protect humans against inflammatory diseases [11] and have been proposed for use against COVID-19 [12]. Similarly, other oligo-based drugs deriving from endogenous ncRNAs have already been approved for clinical trials and can potentially be used for other clinical conditions [7,9]. For example, MRG-110 is an LNA-based antisense oligonucleotide that targets miR-92a-3p and is currently in phase 2 trial as an anti-inflammatory drug to treat patients with cardiovascular complications [7,13].

This study aimed to achieve two goals: first, to identify the existence of endogenous ncRNAs able to interact with the SARS-CoV-2 genome and transcripts that can serve as a template to design RNA-based sequences with antiviral effects; second, to identify and characterize SARS-CoV-2 motifs that might be recognized by selective ncRNAs and therefore, utilized to design RNA-based antiviral drugs and to increase their efficiency.

## 2. Materials and Methods

### 2.1. Dataset

The complete genome of Severe acute respiratory syndrome coronavirus 2 isolate Wuhan-Hu-1 has been downloaded from ENA (MN908947.3) (http://www.ebi.ac.uk/ena/data/view/MN908947.3); 3′- 5′-UTR and the Spike portion have been collected from the same entry. A total of 2656 human miRNA sequences have been downloaded from miRBase (May 2020) [14]. The sequences for the following lncRNAs have been downloaded from RNAcentral [15]: FENDRR, FTX, H19, HOTAIR, MALAT1, MEG3, MHRT, MIAT, NRON, SENCR, lncWDR59, LINC01505, APOA1-AS.

### 2.2. miRNAs and lncRNAs Interaction with SARS-CoV-2 Genome

Three RNA–RNA binding site prediction methods have been considered: IntaRNA [16], RNAplex, and RNAup [17]. This choice is based on two recent comparative studies which highlighted them as the best overall methods [18,19]. These programs have been run with default parameters; exceptions have been made for the maximum matching interaction length, which has been set to the miRNA length and to 100 for lncRNA–RNA matches. Average pair probabilities for locally stable secondary structures, necessary for RNAplex, have been calculated with RNAplfold [17].

Examples of the running commands are:
**IntaRNA**IntaRNA -t <input_file_query> -q <input_file_target> > <output_file>**RNAplex**RNAplfold -W <mirna_length> -u <mirna_length> -O --plex_output < <input_file>RNAplex -l <mirna_length> -q <input_file_query> -t <input_file_target> -a ./**RNAup**RNAup -w <mirna_length> -b -o -3 -5 --interaction_first < <input_file>

The complete procedure, with command line instructions, parameters, and final script for the merging of the results of the three different prediction methods can be found at the following link: https://github.com/lucaparca/mirna_covid (09/02/2021 version).

### 2.3. RNA–RNA Interaction Analysis

Results of the three methods have been analyzed and merged, giving high priority to consensus matches. Match ranges, the overlap of which has been expressed through the Jaccard indexes of the matching residues in both RNAs, and interaction energy (minimum free energy, MFE, expressed in Kcal/mol) are then considered (Supplementary Tables S1 and S2 in the Supplementary Excel file). We considered only match ranges with a Jaccard index threshold of ≥0.8. We considered an MFE threshold of <−20 for miRNA binding sites (BS) predicted at the viral 3′UTR or at the Spike mRNA transcript (3′UTR). We considered an MFE threshold of <−5 for miRNA BS predicted at the viral 5′UTR of SARS-CoV-2 genome, since it is considered a noncanonical site of interaction and available datasets refer mainly to 3′UTR bindings (Supplementary Tables S1 and S2 in the Supplementary Excel file). An interaction propensity threshold of <−15 was arbitrarily set up for lncRNA:RNA interactions. In detail, we calculated the average of all interaction propensities obtained from two out of three methods considering the low interaction propensity of nuclear lncRNAs. Results from RNAup have been excluded since the program returned not reliable data due to the length of lncRNA transcripts. The average of all means was considered as the threshold (interaction propensity <−15).

### 2.4. MiRNAs and SARS-CoV-2 Leader Sequence Motifs Analysis

Enriched motifs have been searched with the MEME suite [20] with default parameters. A logo of the miRNA seed motifs and leader motifs has been generated using WebLogo [17,21] with default parameters.

### 2.5. Logistic Prediction of miRNA-Target Sites Using High Throughput and V-CLIP Studies

miRNA candidates showing BS in the leader sequence were further screened. In particular, miRNAs were analyzed using the STarMir prediction tool [22,23] to implement the logistic prediction, by crosslink of high-throughput miRNA binding data with immune-precipitation (CLIP) studies. The advantage of this additional prediction is that we can incorporate comprehensive thermodynamic, structural, and sequence features. First, for each miRNA candidate interacting with the leader sequence according to RNAup, RNAplex, and IntaRNA analysis, we further analyzed the interaction propensity using the available “5′UTR” filter to screen the high-throughput and V-CLIP datasets. All interactions were confirmed and followed Bartel classification {Bartel, 2009 #25}. Next, we evaluated the target site probability score, ranking the target sites based on their logistic probability, site, and seed access score. Site Access is the measure of structural accessibility as computed by the average probability of a nucleotide being single strand (unpaired) for the nucleotides in the predicted binding site. Seed Access is the measure of structural accessibility as computed by the average of single-stranded probabilities of nucleotides in the target sub-region complementary to the miRNA seed. The potential of nucleation (ΔG_nucl_) and stability (ΔG_hybrid_) of target site annealing was used to measure the total energy change of the hybridization (ΔG_total_) (see Supplementary Table S7 in the Supplementary Excel file for additional details).

We further analyzed the miRNA:leader sequence interaction. Considering the thermodynamics of predicted BS, we calculated the probability of each nucleotide within the leader sequence to be involved in a binding with each miRNA. An unpaired probability value (PU) for each nucleotide below 0.05 corresponded to a significantly high propensity of interaction. In contrast, high PU values correspond to miRNA:RNA loops.

### 2.6. LncRNA Secondary Structures

The interaction propensity between lncRNAs and the Spike transcript, SARS-CoV-2 5′, or 3′UTR was predicted using the RNAfold web tool [24], following the prediction of lncRNA putative secondary structures using the RNAfold web tool. Colors represent base pair probabilities.

## 3. Results

### 3.1. MiRNAs Bind the 5′UTR-Leader Sequence, the 3′UTR of SARS-CoV-2 and the Spike mRNA through Noncanonical Bindings

MiRNAs act as translational repressors as part of an RNA-induced silencing multiprotein complex (RISC) where they target mRNA transcripts at their 3′-UTR with a sequence of 7–8 nucleotides located at their 5’ end, termed miRNA seed [25,26].

Animal miRNAs can also recognize and bind mRNAs at their 5′UTR, acting as enhancers or inhibitors of translation [27,28].

**Figure 1:**
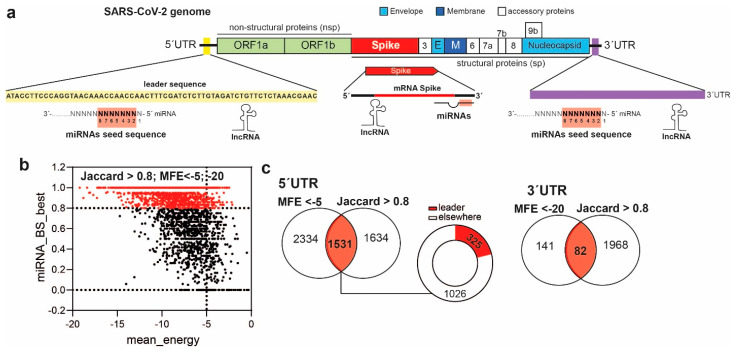
SARS-CoV-2 genomic sequence and miRNA binding sites (BS) identified within the 5′UTR and 3′UTR of SARS-CoV-2 genome. (**a**) Structural representation of SARS-CoV-2 genome with highlighted leader sequence (yellow), 3′UTR (violet), and spike transcript (red) against which we identified (**b**) miRNA BS using 3 RNA:RNA prediction algorithms. Only binding sites with a Jaccard index ≥0.8 and a minimum free energy (MFE) <−5 (for the 5′UTR), or <−20 (for the 3′UTR of viral-RNA and Spike transcript) were considered. Bars: MiRNA_BS_best indicates the Jaccard Index scale, whereas the mean_energy scale bar indicates the MFE. Dashed lines represent the thresholds. (**c**) Representation of miRNA BS identified at the viral 5′UTR, 325 of which were in the leader sequence, and 82 at the 3′UTR.

We analyzed the 3′UTR and the 5′UTR of the viral genome in order to build up a set of miRNA candidates able to interact with the genome of SARS-CoV-2 (Supplementary Tables S1 and S2 in the Supplementary Excel File) and we identified specific miRNA binding sites (BS) on the viral RNA sequence and on the mRNA encoding for the glycoprotein Spike (Figure 1a). A schematic workflow of the entire study including parameters/thresholds used is reported in Supplementary Figure S1. The Spike protein is particularly known to play a pivotal function during infection. Indeed, Spike facilitates SARS-CoV-2 envelope fusion with the cell membrane and the virus endosomal entrance by interacting with the cellular receptor Angiotensin Converting Enzyme 2 (ACE2).

Initially, we screened 2656 human mature miRNA sequences using three separate RNA–RNA binding site prediction methods [16,18]. Although miRNAs are known to interact with the 3′UTR of mRNA transcripts, the 5′UTR of SARS-CoV-2 genome contains a highly conserved sequence of 90 nucleotides, termed the “leader sequence”, that is pivotal for viral transcription and that is used for the identification of all viral subgenomic mRNAs (Figure 1a). Therefore, we considered all miRNA bindings at the viral 5′UTR, with particular interest in those identified within the leader sequence.

After analysis and data merging, we gave high priority to consensus matches among the three different prediction methods. In detail, we considered only match ranges with a Jaccard index threshold of ≥0.8 and with an MFE <−5 for miRNA BS against the 5′UTR, and an MFE<−20 for miRNA BS against the viral and Spike 3′UTR (Figure 1b and Supplementary Tables S3–S5; a detailed description is available in the Methods section).

**Figure 2:**
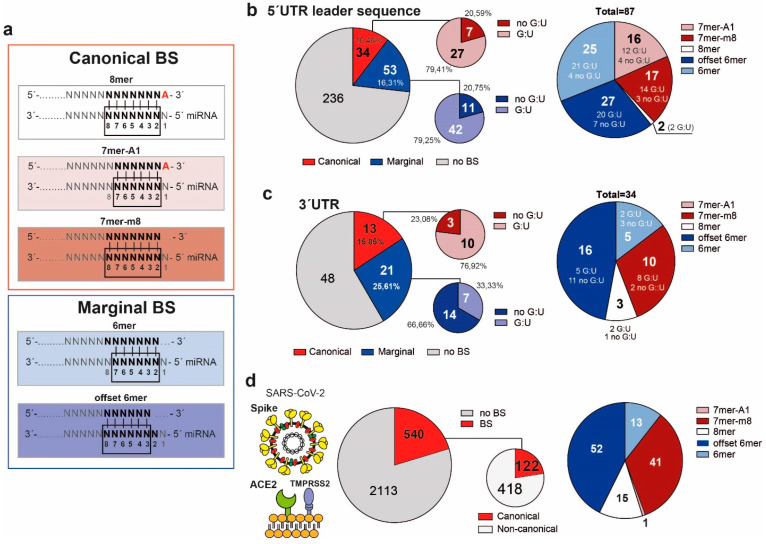
Identified miRNA binding sites (BS) on SARS-CoV-2 leader sequence, genomic 3′UTR, and Spike 3′UTR transcript. (**a**) Bartel classification of miRNA BS according to their seed pairing with mRNA targets, as canonical and marginal. MiRNA seeds involved in the binding with RNA transcript comprise nucleotides (nt) 2–8 (for 8mer, 7mer-m8), nt 2–7 (7mer-A1, 6mer), or nt 3–8 (offset 6mer). (**b**–**d**) MiRNA BS identified and classified according to Bartel classification, and according to classical (G:C) or noncanonical (G:U) seed pairing in the (**b**) viral leader sequence, (**c**) viral genomic 3′UTR, and (**d**) 3′UTR of glycoprotein Spike transcript. The figure represents the SARS-CoV-2 virus and the target cell receptors TMPRSS2 and ACE2, which interact with Spike protein for endocytosis viral entrance. TMPRSS2: transmembrane protease serine subtype 2; ACE2: angiotensin converting enzyme 2.

We identified 1531 miRNA BS against the 5′UTR and 82 miRNA BS against the 3′UTR of SARS-CoV-2 genome (Figure 1c and Supplementary Tables S3–S6). Interestingly, among the set of miRNA BS against the viral 5′UTR, 325 were located within the leader sequence (Figure 1c). According to the Bartel classification, which characterizes functional miRNA–target interactions [25,26], we divided our miRNA BS into two main groups: canonical (7mer-A1, 7mer-m8, and 8mer) and marginal (6mer, and off-set 6mer) (Figure 2a). In particular, all miRNA BS classified as canonical matches comprise G:C and A:U alignments, whereas all matches that comprise G:U alignments are classified as noncanonical [25]. Fifty-three out of 325 miRNA BS against the leader sequence (5′UTR) were classified as marginal BS and 34 as canonical BS (Figure 2b). Twenty-one out of 82 miRNA BS against the 3′UTR were classified as marginal, whereas 13 were classified as canonical BS (Figure 2c).

We previously reported that miRNAs can either engage in noncanonical interactions with proteins, in example inhibiting nuclear caspase-3 [29], or bind “nonclassical targets”, such as lncRNAs, through a more noncanonical and G:U pairing enriched BS [30]. Accordingly, 79% of both canonical and marginal miRNA BS against the 5′UTR leader sequence contain G:U pairs (Figure 2b and Supplementary Table S3).

Within the set of identified miRNA BS against the 3′UTR, G:U pairs were more frequent in canonical compared to marginal BS (Figure 2c and Supplementary Table S4). Conversely, 540 potential miRNA BS identified at the 3′UTR of Spike mRNA lack a G:U pairing (Figure 2d, and Supplementary Table S5).

Irrespective of the genomic region considered, the binding between miRNAs and the leader sequence, the genomic 3′UTR, and the Spike transcript preferentially occur through an offset-6mer binding (Figure 2 and Supplementary Tables S3–S5). Taken together, these data indicate the presence of a conserved and/or preferred binding match within the miRNA seed sequences that might sustain miRNA bindings at the viral genome.

### 3.2. MiRNAs and the Leader Sequence Contain Motifs Increasing miRNA: Viral RNA Selectivity

**Figure 3:**
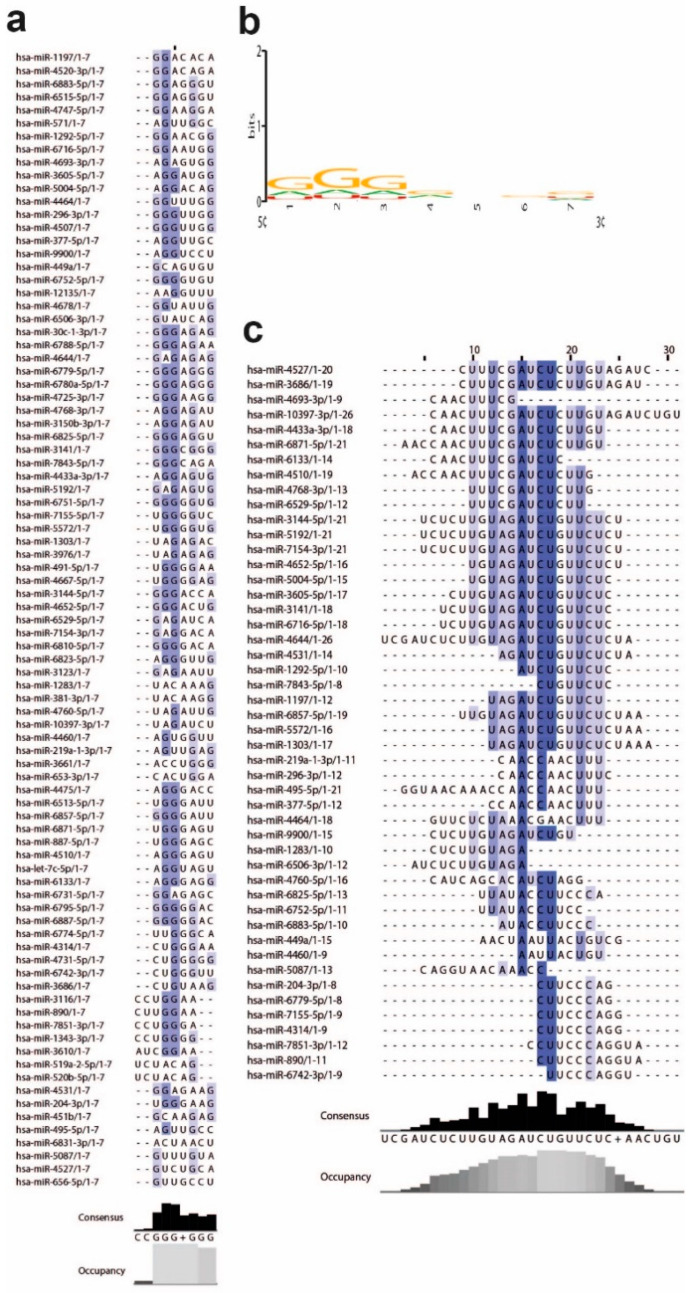
GGG and leader sequence motifs identified using RNA:RNA algorithms against all miRNA candidates. (**a**,**b**) miRNAs targeting the leader sequence of SARS-CoV-2 5′UTR genome contained the GGG motif in their seed. (**a**) The GGG motif is colored in blue, (**b**) and the MEME representation is indicated in yellow. The consensus and occupancy are reported at the bottom of the list. (**c**) List of miRNAs matching the three motifs identified in the SARS-CoV-2 genome. The blue indicates the position of the motifs and the recurrency. The consensus and occupancy are reported at the bottom of the list.

Irrespective of the type of BS classification, miRNAs that bound the leader sequence through noncanonical and G:U paired BS were characterized by a “GGG” nucleotide (nt) motif in their seed (nt 2, 3, 4) (Figure 3a,b and Supplementary Table S6). The “GGG” motif was absent in the seed of miRNAs binding the genomic 3′UTR or the Spike transcript. We did not identify conserved motifs among all 2656 human miRNAs, nor references reporting conserved motifs in miRNA seed sequences that might be used to increase miRNA selectivity against their mRNA targets. Thus, these data indicate the existence of selective miRNAs that can recognize the viral leader sequence through a GGG motif, which may stabilize miRNA noncanonical binding at the viral genome.

**Figure 4:**
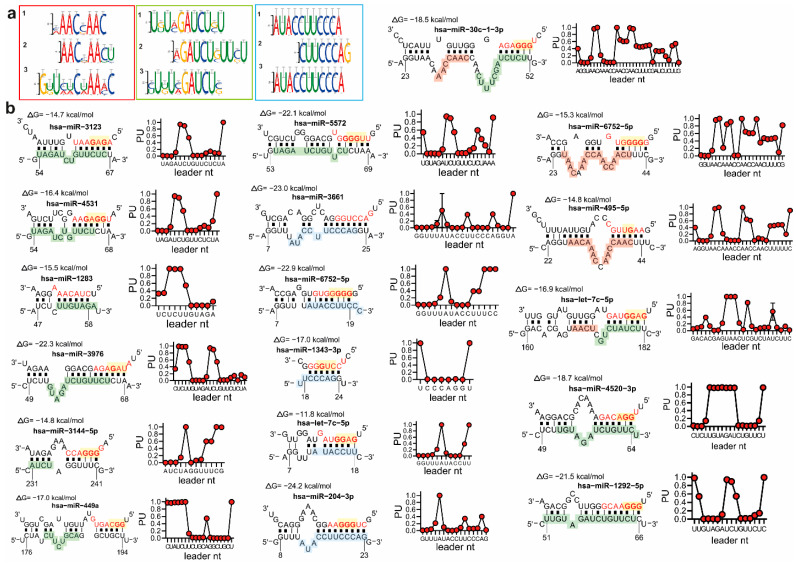
Detailed motifs identified at the SARS-CoV-2 5′UTR leader sequence and thermodynamic evaluation of their interaction with miRNAs with a GGG motif in their seed. (**a**) Three leader motifs identified with RNAup (1), IntaRNA (2), and RNAplex (3). (**b**) Representation of miRNAs and their binding to the three motifs identified in the viral leader sequence. In yellow, the GGG motif. The three motifs are colored like in panel (**a**): AACnAAC (red), UnUnGAUCUnU (green), and AUACCUUCCCA motifs (light blue). Total free energy is represented as ΔG and expressed in kcal/mol. The thermodynamic, structural, and, therefore, reliable miRNA:RNA interactions analyzed using the StarMir algorithm are reported closed to each gmiRNA:RNA graphical representation. Data are represented as the probability of each nucleotide to be unpaired (PU), considering thermodynamic and structural features analyzed. The X axis of the graph reports the nucleotides (nt) of the viral leader sequence involved in the binding with miRNAs.

Analysis of the enriched motifs in the leader sequence of SARS-CoV-2 identified three consensus motifs recognized by miRNAs with a GGG-enriched seed and containing G:U pairs (Figure 3c and Figure 4a,b and Supplementary Table S6). In particular, we identified an “AACnAAC”, an “AUACCUUCCA”, and an “nUnGAUCUnU” motif recognized by the miRNA seed sequence, in particular by the nucleotides 3–8 of miRNA seeds. (Figure 3c, Figure 4a, and Figure S2, and Supplementary Table S6). Interestingly, GGG-enriched miRNA candidates recognized more than one motif (Figure 4b and Figure S2, and Supplementary Table S6).

Considering the miRNAs targeting the leader sequence in the viral genome (Supplementary Table S6), we predicted their targets using miRDB [31] (using a score threshold of 90), which contains the miRNA target predictions made with MirTarget [32]. We then considered the data of the genes differentially expressed in COVID-19 patients [33] and intersected them with the list of genes targeted by the miRNAs (Supplementary Table S7 in the Supplementary Excel File). These genes are significantly differentially expressed compared to a set of random genes of equal size (Mann–Whitney U *p*-value < 0.01, two-sided) (Supplementary Figure S2 and Supplementary Table S7 in the Supplementary Excel File). These genes were enriched in NO production in the heart, regulation of the cytoskeleton, mitotic spindle, circadian entrainment, and generally to the cGMP-PKG and WNT pathways, the neuronal system, and smooth muscle cell dysfunction in pulmonary hypertension. These genes are also involved in chronic bronchitis, atrial fibrillation, thoracic aortic aneurysm, aortic dissection, and arterial stiffness, which are conditions related to COVID-19 patients.

The next step was the logistic prediction of miRNA:leader RNA folding and pairing using the StarMir algorithm [22], based on available high-throughput and V-CLIP datasets to provide thermodynamic, structural, and, therefore, reliable miRNA:RNA interactions. This prediction provides an output value termed “probability of unpaired” (PU), which is the probability for each nucleotide of the leader sequence to bind to a miRNA according to miRNA:leader-sequence thermodynamic features (Supplementary Table S8 in the Supplementary Excel File). Accordingly, the logistic prediction confirmed the propensity of GGG-enriched miRNAs to selectively interact with leader-enriched motifs (Figure 4b and Figure S3). The logistic prediction identified the presence of thermodynamically stable miRNA:RNA BS, as well as of thermodynamically unstable BS, and therefore, of an improbable binding, as in the example for miR-5572 (Figure 4b and Figure S3).

In order to provide a list of the best candidates that might be considered designing RNA-based antiviral drugs, we screened the currently available literature to select only the miRNAs involved in viral response and pulmonary and cardiovascular diseases. Among the thermodynamically stable ones, we identified 35, 16, and 44 miRNA candidates able to bind the leader sequence, the viral 3′UTR, and the Spike mRNA, respectively (Table 1 and Table S9). Fifteen miRNAs targeting the leader sequence modulate viral replication upon infection in humans. As an example, miR-3661 is directly involved in the formation of SARS-CoV-2 proteins in lung [34], whereas miR-3145-5p and let-7c-5p inhibit viral H1N1-deriving protein synthesis in chronic obstructive pulmonary disease (COPD) [35,36]. Seven of the identified miRNAs, such as miR-1292, miR-219a, miR-30c-1, miR-5572, miR-451b, miR-491, and miR-6887, are involved in arthritis and osteonecrosis [37,38]. However, miR-5572 was excluded by our thermodynamic analysis. MiR-449a [39], miR-4531 [40], and miR-204 [41] are involved in pulmonary arterial hypertension (PAH), asthma, COPD, and pulmonary fibrosis. Airway epithelial cells affected by mucin overproduction have been reported to be highly enriched in miR-6752 [42]. Similarly, miR-4531 is upregulated in children with asthma, and miR-4520-3p is associated with familial Mediterranean fever [43]. Hence, miRNAs targeting the leader sequence of SARS-CoV-2 might partly explain the increased susceptibility of certain patients showing familial or previous complications.

Four miRNAs have been independently identified in a previous work [44]: let-7c-5p, miR-1183, miR-4500 (binding the Spike 3′UTR region), and miR-1197 (binding the leader sequence).

Binding sites for four additional miRNAs on the 5′UTR of the viral genome have been found in another work [45]. Interestingly, the authors of this work tried to identify binding sites for RNA-binding proteins (RBP) on the viral genome. We could observe that binding sites predicted for the interaction of two RBP (PCBP2 and SRSF1) overlap with some of the binding sites that we predicted for some of the miRNAs on the viral leader sequence: the binding site of PCBP2 on the viral leader sequence overlaps with the binding sites of hsa-miR-204-3p, hsa-miR-1343-3p, hsa-miR-3661, hsa-miR-30c-1-3p, hsa-miR-449a, and hsa-miR-6752-5p, while the binding site of SRSF1 RBP overlaps with the binding site of hsa-miR-219a-5p. These observations open up an additional layer of regulation mechanisms for the viral infection, with different non-coding RNAs and other proteins able to bind RNA possibly competing for the same binding sites. This could provide additional key targets to consider when designing novel therapeutic strategies.

### 3.3. lncRNAs H19, LINC01505, and Fendrr Interact with SARS-CoV-2 and Spike mRNA

Except for a new class of functional RNAs, i.e., SRA [70], that are able to encode a protein, most lncRNA transcripts are not translated into proteins. LncRNAs are around 200 nucleotides or more in length, as compared to small non-coding RNAs. Similar to miRNAs, lncRNAs can interact with RNA, DNA, and proteins, and form RNA–RNA, RNA–DNA, and RNA–protein complexes, leading to the regulation of gene expression via multiple mechanisms, including modulation of transcription, mRNA stability, and translation [71].

**Figure 5:**
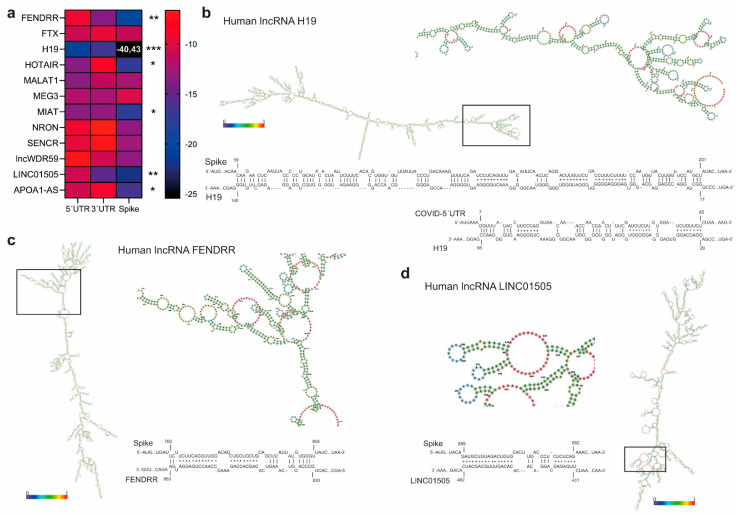
Interaction propensity of lncRNAs involved in pulmonary arterial hypertension, antiviral response, and inflammatory diseases. (**a**) Heat map of lncRNA interaction propensity with SARS-CoV-2 5′UTR, 3′UTR, and with Spike mRNA using IntaRNA, RNAup, and RNAplex. Significantly binding sites for Spike were identified for cytoplasmic (**b**) lncRNAs H19 (and for 5′UTR), (**c**) FENDRR, and (**d**) LINC01505. Nuclear lncRNAs were used as negative control. Rectangles represent the zoomed view of lncRNA interaction loops. LncRNA minimum free energy secondary structures were predicted using RNAfold web tool. Colors represent base pair probabilities. * *p* < 0.05; ** *p* < 0.01; *** *p* < 0.001.

Based on the available literature, we selected 12 potential human lncRNAs with a function related to pulmonary hypertension and cardiovascular and inflammatory diseases, to be considered as candidates for a functional interaction with SARS-CoV-2 in the cytoplasm. (Figure 5a). We also included six lncRNAs with a reported function in the nucleus, i.e., DNA target or as epigenetic regulators of gene expression, as negative controls within our analysis. According to their nuclear localization, we assumed that interaction with SARS-CoV-2 transcripts was unlikely. According to the same computational approach adopted for miRNA BS identification, and considering lncRNA complex secondary structures, lncRNA H19 showed the highest and most significant interaction propensity (IE) with the SARS-CoV-2 5′UTR (−20.82) and Spike mRNA (−40.43) (Figure 5b). Except for MIAT (IE -17) and APOA1-AS (IE -16) that showed a mild binding propensity for Spike mRNA, all nuclear lncRNAs lacked a potential viral–RNA interaction.

Three LncRNAs, such as FENDRR, HOTAIR, and LINC01505, were found to potentially interact with Spike mRNA. Given that the secondary structure of HOTAIR has been experimentally determined [72], we were able to map its interacting regions with the Spike mRNA in Domain 2, elements H16–H21, and in Domain 4, elements H50–52. LINC01505 also showed a binding propensity for SARS-CoV-2 3′UTR (Figure 5c,d). Notably, H19 has been found to promote the pathogenesis of pulmonary arterial hypertension (PAH) [73], suggesting that it might also contribute to SARS-CoV-2 acute pulmonary injury.

## 4. Discussion

Our computational study provides an original starting background to facilitate the identification of novel effective RNA-based anti-COVID-19 therapies as an expedient alternative to “conventional” anti-inflammatory and antiviral treatments.

We performed a thorough screening of the viral genome in order to identify sites of possible interaction with human non-coding RNAs by a computational analysis.

According to our analysis, we propose a set of miRNAs and lncRNAs as candidates to negatively modulate viral genome expression, possibly resulting in a reduction in SARS-CoV-2 infectivity.

Notably, we identified miRNA BS able to bind the mRNA encoding for the glycoprotein Spike, a protein playing a central role in the infection as it facilitates SARS-CoV-2 envelope fusion with the cell membrane and the virus endosomal entrance by interacting with the cellular receptor ACE2. Although ACE2 has been proposed as a target for antiviral treatments [74], its fundamental protective role against acute lung failure [75] led us to hypothesize that the identification of putative miRNA and lncRNA BS on Spike mRNA may represent a more effective mechanism, with less side-effects, to prevent SARS-CoV-2 transcription.

As reported in the results section, we identified a “GGG” motif in the seed of miRNAs binding the viral leader sequence through noncanonical and G:U paired BS. Conversely, this motif was absent in miRNA seeds binding the genomic 3′UTR or the Spike transcript. Indeed, we did not identify conserved motifs among all 2656 human miRNAs, nor references reporting conserved motifs in miRNAs seed sequences that might be used to increase miRNA selectivity against their mRNA targets. The presence of a specific group of miRNAs with UGUGU able to activate targets associated to interferon induction and carcinogenesis has been previously reported [76]. The same paper also reports that G:U-rich miRNAs are involved in the regulation of neurogenesis, and purine/pyrimidine-rich miRNAs are involved in RNAs transport and/or degradation.

The logistic prediction of miRNA:leader RNA folding and pairing using the StarMir algorithm indicated that leader-enriched motifs might increase the stability of GGG-enriched miRNAs that target the SARS-CoV-2 genome through noncanonical bindings. These motifs might be promising candidate sites to design antiviral RNA-based drugs.

The COVID-19 global pandemic and following re-emergence events subjected the public health system to a tremendous crisis. Considering the dramatic respiratory failure to which patients severely affected by COVID-19 are subjected and the potential lethality, priority is to be given in reducing viral-related complications [5].

Endogenous ncRNAs, known modulators of viral and inflammatory mechanisms, can represent a class of molecules offering an evolutionary and adaptive advantage in humans and a more attractive and promising strategy against COVID-19. As observed in this and in previous works, an additional layer of viral infection regulation and modulation is provided by the possible crosstalk between different non-coding RNAs competing for the same binding sites and with RNA binding proteins. The interplay among these molecules can be perturbed and taken advantage of by the viral infection, providing interesting and novel targets for the design of novel therapies. However, ncRNA-based drug specificity against the viral genome may represent an obstacle when modulating endogenous ncRNAs. Here, we have identified and characterized selective motifs located in the SARS-CoV-2 genome, and in the seed of certain miRNAs, which may represent the background to generate viral-selective RNA-based drugs with unwanted side effects.

LNA-based anti-miRs are already reported to efficiently target endogenous miRNAs in vivo [77], and can be easily modified to increase their stability [78,79]. Accordingly, LNA-based oligosequences are usually modified or combined with deoxyribonucleotides within the RNA sequence to create more stable and efficient LNA-DNA mixmers. Moreover, to favor their administration in humans and to increase their availability and overall, their efficiency, LNA-based oligosequences are encapsulated into liposomes and delivered as lipid nanoparticles [80,81]. This modification and administration have been successfully used for mRNA-based vaccines that reach phase trials with promising efficacy [80]. LNA-based anti-miRs are administered in vivo using the same principle, i.e., anti miR-92a-3p [13], supporting our goal to promote viral-selective RNA-based drugs against COVID-19 complications.

In this work, we consider lncRNAs as well, which can interplay with miRNAs, for example by acting as sponges of miRNAs, and as we demonstrated in this work, can bind to the viral genome and Spike transcript. Our data provide novel insights on lncRNAs as additional ncRNA molecules with the potential to interact with the SARS-CoV-2 genome and Spike transcript, and therefore, be candidates to design lncRNA-based oligosequences, in example circular RNAs. Indeed, lncRNAs and cirRNAs are promising novel RNA-based therapeutic strategies, already used in patients with heart failure as well as on wound healing [82]. Overall, in silico predictions and interactions are considered a crucial starting point to select promising candidates [82].

Although strategies such as monoclonal antibodies against Spike [83], or inhibitors of viral enzymes, are much more advanced in the treatment of viral infections, and specifically of COVID-19, the high mutation rate in Spike proteins [3] must be considered from a long-term perspective. Hence, RNA-based therapies matching untranslated and conserved parts of the SARS-CoV-2 genome, such as the leader sequence, might be a promising alternative.

It is an important note to readers to consider that our study is not claiming efficacy/potential for a specific treatment. The authors acknowledge that a different, more integrated experimental approach is indispensable to design a clinically testable compound.

## Figures and Tables

**Table 1:** Role of main miRNA candidates binding the leader sequence of SARS-CoV-2.

miRNAs	Reported Function	Reference
hsa-miR-1283	Endothelial vascular injury	[46]
hsa-miR-495-5p	Inhibits vascular remodeling and angiogenesis in PAH	[47]
hsa-miR-1303	Regulate the autophagy process in mycobacteria infection	[48]
hsa-miR-204-3p	Promoter of PAH	[41]
hsa-miR-6529-5p	Novel potential tissue specific biomarker in cattle	[49]
hsa-miR-1343-3p	Attenuate fibrosis in fibrotic lung disease/microvesicle	[50,51]
hsa-miR-3661	Direct involvement with SARS-CoV-2 proteins from lung biopsy	[34]
hsa-miR-381-3p	Deregulated in lung adenocarcinoma	[52]
hsa-miR-3976	Regulates apoptosis in hosts after microbial infection	[53]
hsa-miR-520b-5p	Inhibits NSCLC	[54]
hsa-miR-3144-5p	Interact with viral proteins	[55]
hsa-miR-4652-5p	Lung cancer expressed miRNA	[56]
hsa-miR-6857-5p	Prognostic of viral-related cervical cancer/marker of NSCLC	[57]
hsa-miR-377-5p	Promote fibronectin production/inhibits lung cell proliferation	[58,59]
hsa-miR-1292-5p	Inhibitor of osteogenic differentiation, promotes osteoporosis	[60]
hsa-miR-219a	Arthritis/NSCLC	[37]
hsa-miR-30c-1-3p	Positive bone development/promotes viral infection	[61,62]
hsa-miR-449a	Inhibits pulmonary fibrosis	[39]
hsa-miR-5572	Upregulated in osteonecrosis femoral head	[38]
hsa-miR-6752-5p	Highly expressed in airway epithelial cells/mucin overproduction	[42]
hsa-miR-4531	Upregulated in children with asthma	[40]
hsa-miR-6831-3p	Anti-atherogenic/PAH	[42,63]
hsa-miR-377-5p	Inhibits lung cancer cell proliferation	[59]
hsa-miR-3123	Negative correlation with survival of COPD patients	[64]
hsa-miR-3150b-3p	Inhibits cell proliferation in NSCLC patients	[65]
hsa-miR-451b	Inhibits osteosarcoma lung metastasis	[66]
hsa-miR-4520-3p	Associated with FMF-related mutations	[43]
hsa-miR-491-5p	Inhibits osteosarcoma lung metastasis	[66]
hsa-miR-6515	Contributes to lncRNA H19-mediated lung cancer metastasis	[67]
hsa-let-7c-5p	Inhibits H1N1 protein synthesis/anti-inflammatory role in COPD	[33,36,68]
hsa-miR-6887-5p	Inhibits squamous cell carcinoma cell growth	[69]

PAH—pulmonary arterial hypertension; NSCLC—non-small-cell lung carcinoma; COPD—chronic obstructive pulmonary disease; FMF—familial Mediterranean fever.

## Data Availability

The data herein presented are available in this article, in the Supplementary Material and in GitHub.

## Supplementary Materials

The following are available online at https://www.mdpi.com/2311-553X/7/1/14/s1, Supplementary Excel File with the Supplementary Tables S1, S2, S7, and S8. Supplementary Table S3: miRNA candidates with binding sites against SARS-CoV-2 leader sequence, Supplementary Table S4: miRNA candidates with BS against SARS-CoV-2 3′UTR, Supplementary Table S5: miRNA candidates with canonical BS against Spike mRNA (3′UTR), Supplementary Table S6: SARS-CoV-2 consensus motifs identified at the 5′UTR leader sequence, Supplementary Table S9: Role of miRNA candidates against Spike transcript and the 3′UTR of SARS-CoV-2, Supplementary Figure S1. Schematic workflow for the entire study including the parameters/threshold used at each step, Supplementary Figure S2. Distribution of the differential expression values of genes targeted by the miRNAs targeting the leader sequence of the SARS-CoV-2, Supplementary Figure S3. GGG motif and leader motifs from selected miRNA candidates interacting with the leader sequence of SARS-CoV-2.
