# Supplementary material for: MicroRNAs and Long Non-Coding RNAs as Potential Candidates to Target Specific Motifs of SARS-CoV-2
Source: Noncoding RNA. 2021 Feb 18;7(1):14. doi: 10.3390/ncrna7010014 (PMC7931055; doi:10.3390/ncrna7010014)
Supplement: Supplementary file 1 [file ncrna-07-00014-s001.zip › ncrna-1091210-supplementary.v10/ncRNA-1091210 Supplementary material-2.pdf]

non-coding RNA

MDPI

Article

MicroRNAs and Long Non-Coding RNAs as Potential Candidates to Target Specific Motifs of SARS-CoV-2

Lucia Natarelli [1,*,†], Luca Parca [2,†], Tommaso Mazza [2], Christian Weber [1,3,4,5,*], Fabio Virgili [6] and Deborah Fratantonio [7]

[1] Institute for Cardiovascular Prevention (IPEK), Ludwig-Maximilians-Universität (LMU), 800336 Munich, Germany
[2] IRCCS Casa sollievo della Sofferenza, Laboratory of Bioinformatics, 71013 San Giovanni Rotondo (FG), Italy; l.parca@css-mendel.it (L.P.); t.mazza@css-mendel.it (T.M.)
[3] German Center for Cardiovascular Research (DZHK), partner site Munich Heart Alliance, 80336 Munich, Germany
[4] Department of Biochemistry, Cardiovascular Research Institute Maastricht (CARIM), Maastricht University, 6200 MD Maastricht, The Netherlands
[5] Munich Cluster for Systems Neurology (SyNergy), 81377 Munich, Germany
[6] Council for Agricultural Research and Economics, Research Center for Food and Nutrition, 00178 Rome, Italy; fabio.virgili@crea.gov.it
[7] Biotechnology and Biopharmaceutics, Department of Biosciences, University of Bari Aldo Moro, 70125 Bari, Italy; deborah.fratantonio@uniba.it
* Correspondence: lnatarel@med.lmu.de (L.N.); christian.weber@med.uni-muenchen.de (C.W.)
† These authors contributed equally to this paper.

Citation: Natarelli, L.; Parca, L.; Mazza, T.; Weber, C.; Virgili, F.; Fratantonio, D. MicroRNAs and Long Non-Coding RNAs as Potential Candidates to Target Specific Motifs of SARS-CoV-2. *Non-coding RNA* **2021**, *7*, x. https://doi.org/10.3390/xxxxx

Academic Editor: George A Calin

Received: 12 January 2021
Accepted: 16 February 2021
Published: 18 February 2021

Publisher's Note: MDPI stays neutral with regard to jurisdictional claims in published maps and institutional affiliations.

Copyright: © 2021 by the authors. Licensee MDPI, Basel, Switzerland. This article is an open access article distributed under the terms and conditions of the Creative Commons Attribution (CC BY) license (http://creativecommons.org/licenses/by/4.0/).

Abstract: The respiratory system is one of the most affected targets of SARS-CoV-2. Various therapies have been utilized to counter viral-induced inflammatory complications, with diverse success rates. Pending the distribution of an effective vaccine to the whole population and the achievement of a "herd immunity", the discovery of novel specific therapies is to be considered a very important objective. Here we report a computational study demonstrating the existence of target motifs in SARS-CoV-2 genome suitable for a specific binding with endogenous human micro and long non-coding RNAs (miRNAs and lncRNAs, respectively), that can be therefore considered a conceptual background for the development of miRNA-based drugs against COVID-19. SARS-CoV-2 genome contains 3 motifs in the 5′UTR leader sequence recognized by selective nucleotides within the seed sequence of specific human miRNAs. The seed of 57 microRNAs contained a "GGG" motif that promoted leader sequence-recognition, primarily through offset-6mer sites able to promote microRNAs noncanonical binding to viral RNA. Similarly, lncRNA H19 binds to the 5′UTR of the viral genome and, more specifically, to the transcript of viral gene Spike, which has a pivotal role in viral infection. Notably, some of the non-coding RNAs identified in our study as candidates for inhibiting SARS-CoV-2 gene expression have already been proposed against diverse viral infections, pulmonary arterial hypertension, and related diseases.

Keywords: oligosequences; SARS-CoV-2; COVID-19; target therapy; non-coding RNAs

1. Supplementary Materials

Table S3. miRNA candidates with binding sites against SARS-CoV-2 leader sequence.

miRNA	BS type	GU	miRNA	BS type	GU
hsa-miR-1197	6mer	1	hsa-miR-6825-5p	7mer-m8	1
hsa-miR-1283	6mer	-	hsa-miR-7843-5p	7mer-m8	2
hsa-miR-1292-5p	6mer	1	hsa-miR-887-5p	7mer-m8	-
hsa-miR-219a-1-3p	6mer	1	hsa-miR-3686	7mer-m8	2
hsa-miR-3605-5p	6mer	2	hsa-miR-377-5p	8mer	2
hsa-miR-4433a-3p	6mer	1	hsa-miR-4314	7mer-m8	1
hsa-miR-4460	6mer	3	hsa-miR-204-3p	7mer-m8	-
hsa-miR-4475	6mer	1	hsa-miR-296-3p	7mer-m8	2
hsa-miR-449a	6mer	2	hsa-miR-4464	7mer-m8	1
hsa-miR-4510	6mer	2	hsa-miR-4768-3p	7mer-m8	1
hsa-miR-4652-5p	6mer	2	hsa-miR-5004-5p	7mer-m8	1
hsa-miR-4678	6mer	-	hsa-miR-571	7mer-m8	1
hsa-miR-4693-3p	6mer	1	hsa-miR-6529-5p	7mer-m8	-
hsa-miR-4760-5p	6mer	1	hsa-miR-6716-5p	7mer-m8	2
hsa-miR-495-5p	6mer	-	hsa-miR-6779-5p	7mer-m8	1
hsa-miR-5572	6mer	2	hsa-miR-4531	8mer	1
hsa-miR-6513-5p	6mer	1	hsa-miR-3123	8mer	1
hsa-miR-653-3p	6mer	1	hsa-miR-12135	offset 6mer	1
hsa-miR-6742-3p	6mer	-	hsa-miR-1343-3p	offset 6mer	-
hsa-miR-6752-5p	6mer	2	hsa-miR-3150b-3p	offset 6mer	1
hsa-miR-6774-5p	6mer	1	hsa-miR-3610	offset 6mer	1
hsa-miR-6788-5p	6mer	1	hsa-miR-3661	offset 6mer	-
hsa-miR-6857-5p	6mer	2	hsa-miR-381-3p	offset 6mer	-
hsa-miR-7155-5p	6mer	1	hsa-miR-3976	offset 6mer	-
hsa-miR-9900	6mer	1	hsa-miR-4507	offset 6mer	2
hsa-let-7c-5p	6mer	2	hsa-miR-451b	offset 6mer	1
hsa-miR-1303	7mer-A1	-	hsa-miR-4520-3p	offset 6mer	1
hsa-miR-3116	7mer-A1	1	hsa-miR-4667-5p	offset 6mer	2
hsa-miR-4644	7mer-A1	2	hsa-miR-4725-3p	offset 6mer	-
hsa-miR-4747-5p	7mer-A1	-	hsa-miR-4731-5p	offset 6mer	1
hsa-miR-6831-3p	7mer-A1	2	hsa-miR-491-5p	offset 6mer	1
hsa-miR-6883-5p	7mer-A1	1	hsa-miR-519-2-5p	offset 6mer	-
hsa-miR-7154-3p	7mer-A1	2	hsa-miR-520b-5p	offset 6mer	-
hsa-miR-5087	7mer-A1	-	hsa-miR-6133	offset 6mer	2
hsa-miR-7851-3p	7mer-A1	-	hsa-miR-6506-3p	offset 6mer	1
hsa-miR-890	7mer-A1	1	hsa-miR-6515-5p	offset 6mer	1
hsa-miR-5192	7mer-A1	2	hsa-miR-656-5p	offset 6mer	1
hsa-miR-4527	7mer-A1	2	hsa-miR-6731-5p	offset 6mer	2
hsa-miR-6751-5p	7mer-A1	1	hsa-miR-6795-5p	offset 6mer	3
hsa-miR-6871-5p	7mer-A1	2	hsa-miR-6810-5p	offset 6mer	2
hsa-miR-3141	7mer-m8	3	hsa-miR-6823-5p	offset 6mer	2
hsa-miR-10397-3p	7mer-m8	1	hsa-miR-6887-5p	offset 6mer	3
hsa-miR-6780a-5p	7mer-m8	1	hsa-miR-30c-1-3p	offset-6mer	2
hsa-miR-6825-5p	7mer-m8	1	hsa-miR-3144-5p	offset-6mer	2

Table S4. miRNA candidates with BS against SARS-CoV-2 3´UTR.

miRNA	BS type	GU	miRNA	BS type	GU
hsa-miR-1183	6mer	-	hsa-miR-12114	offset-6mer	2
hsa-miR-449c-5p	6mer	-	hsa-miR-1304-3p	offset-6mer	-
hsa-miR-4710	6mer	1	hsa-miR-130b-5p	offset-6mer	2
hsa-miR-8071	6mer	1	hsa-miR-1471	offset-6mer	-
hsa-miR-885-3p	6mer	-	hsa-miR-4456	offset-6mer	-
hsa-miR-1204	7mer-m8	-	hsa-miR-4525	offset-6mer	-
hsa-miR-183-5p	7mer-m8	1	hsa-miR-4700-5p	offset-6mer	-
hsa-miR-22-5p	7mer-m8	1	hsa-miR-4721	offset-6mer	-
hsa-miR-3605-3p	7mer-m8	1	hsa-miR-486-3p	offset-6mer	1
hsa-miR-384	7mer-m8	1	hsa-miR-5008-3p	offset-6mer	2
hsa-miR-4328	7mer-m8	2	hsa-miR-5010-5p	offset-6mer	-
hsa-miR-4446-3p	7mer-m8	2	hsa-miR-6507-3p	offset-6mer	-
hsa-miR-595	7mer-m8	1	hsa-miR-6732-5p	offset-6mer	-
hsa-miR-6803-5p	7mer-m8	1	hsa-miR-6778-3p	offset-6mer	-
hsa-miR-6890-5p	7mer-m8	-	hsa-miR-6796-3p	offset-6mer	-
hsa-miR-5093	8mer	1	hsa-miR-92a-2-5p	offset-6mer	1
hsa-miR-6835-5p	8mer	1	hsa-miR-6867-3p	8mer	-

Table S5. miRNA candidates with canonical BS against Spike mRNA (3´UTR).

miRNA	BS type	miRNA	BS type	miRNA	BS type
hsa-miR-1298-3p	6mer	hsa-miR-4690-3p	7mer-m8	hsa-miR-646	offset-6mer
hsa-miR-4474-3p	6mer	hsa-miR-604	7mer-m8	hsa-miR-513b-5p	offset-6mer
hsa-miR-3672	6mer	hsa-miR-4278	7mer-m8	hsa-miR-378a-5p	offset-6mer
hsa-miR-7157-5p	6mer	hsa-miR-3155b	7mer-m8	hsa-miR-4802-5p	offset-6mer
hsa-miR-4330	6mer	hsa-miR-6501-3p	7mer-m8	hsa-miR-143-3p	offset-6mer
hsa-miR-624-3p	6mer	hsa-miR-3939	7mer-m8	hsa-miR-555	offset-6mer
hsa-miR-4524a-5p	6mer	hsa-miR-4733-3p	7mer-m8	hsa-miR-425-5p	offset-6mer
hsa-miR-548an	6mer	hsa-miR-627-5p	7mer-m8	hsa-miR-1287-3p	offset-6mer
hsa-miR-939-3p	6mer	hsa-miR-6890-3p	7mer-m8	hsa-miR-1285-5p	offset-6mer
hsa-miR-3145-5p	6mer	hsa-miR-196a-3p	7mer-m8	hsa-miR-7977	offset-6mer
hsa-miR-605-3p	6mer	hsa-miR-3686	7mer-m8	hsa-miR-6715b-5p	offset-6mer
hsa-miR-660-3p	6mer	hsa-miR-4292	7mer-m8	hsa-miR-651-3p	offset-6mer
hsa-miR-548aq-5p	6mer	hsa-miR-4799-3p	7mer-m8	hsa-miR-620	offset-6mer
hsa-miR-1298-3p	6mer	hsa-miR-632	7mer-m8	hsa-miR-3127-3p	offset-6mer
hsa-miR-4474-3p	6mer	hsa-miR-5088-3p	7mer-m8	hsa-miR-664a-5p	offset-6mer
hsa-miR-3672	6mer	hsa-miR-4306	7mer-m8	hsa-miR-320b	offset-6mer
hsa-miR-563	7mer-A1	hsa-miR-4438	8mer	hsa-miR-5003-5p	offset-6mer
hsa-miR-3155a	7mer-m8	hsa-miR-3616-3p	8mer	hsa-miR-3622a-3p	offset-6mer
hsa-miR-648	7mer-m8	hsa-miR-4694-3p	8mer	hsa-miR-4793-3p	offset-6mer
hsa-miR-23a-3p	7mer-m8	hsa-miR-3606-5p	8mer	hsa-miR-320c	offset-6mer
hsa-miR-4722-5p	7mer-m8	hsa-miR-219b-5p	8mer	hsa-miR-661	offset-6mer
hsa-miR-5006-5p	7mer-m8	hsa-miR-581	8mer	hsa-miR-603	offset-6mer
hsa-miR-4457	7mer-m8	hsa-miR-12125	8mer	hsa-miR-1226-3p	offset-6mer
hsa-miR-4678	7mer-m8	hsa-miR-4500	8mer	hsa-miR-3138	offset-6mer
hsa-miR-554	7mer-m8	hsa-miR-548av-5p	8mer	hsa-miR-4742-5p	offset-6mer
hsa-miR-492	7mer-m8	hsa-miR-4641	8mer	hsa-miR-7515	offset-6mer
hsa-miR-4499	7mer-m8	hsa-miR-19a-3p	8mer	hsa-miR-196a-1-3p	offset-6mer
hsa-miR-135a-5p	7mer-m8	hsa-miR-342-3p	8mer	hsa-miR-515-3p	offset-6mer
hsa-miR-3193	7mer-m8	hsa-miR-7705	8mer	hsa-miR-410-3p	offset-6mer
hsa-miR-4327	7mer-m8	hsa-miR-3143	8mer	hsa-miR-526b-5p	offset-6mer
hsa-miR-6816-3p	7mer-m8	hsa-miR-3085-5p	8mer	hsa-miR-6881-5p	offset-6mer
hsa-miR-324-3p	7mer-m8	hsa-miR-4735-3p	offset-6mer	hsa-miR-5000-3p	offset-6mer
hsa-miR-1207-3p	7mer-m8	hsa-miR-6088	offset-6mer	hsa-miR-335-5p	offset-6mer
hsa-miR-484	7mer-m8	hsa-miR-320d	offset-6mer	hsa-miR-6848-3p	offset-6mer
hsa-miR-4662a-5p	7mer-m8	hsa-miR-1183	offset-6mer	hsa-miR-205-3p	offset-6mer
hsa-miR-708-5p	7mer-m8	hsa-miR-4732-3p	offset-6mer	hsa-miR-4768-3p	offset-6mer
hsa-miR-6124	7mer-m8	hsa-miR-4797-5p	offset-6mer	hsa-miR-34b-5p	offset-6mer
hsa-miR-1293	7mer-m8	hsa-miR-4778-5p	offset-6mer	hsa-miR-6529-3p	offset-6mer
hsa-miR-6895-3p	7mer-m8	hsa-miR-490-3p	offset-6mer	hsa-miR-6776-5p	offset-6mer
hsa-miR-4421	7mer-m8	hsa-miR-6508-5p	offset-6mer	hsa-miR-616-5p	offset-6mer
hsa-miR-2278	7mer-m8	hsa-miR-200b-3p	offset-6mer	hsa-miR-6727-3p	offset-6mer
hsa-miR-4668-5p	7mer-m8				

Table S6. SARS-CoV-2 consensus motifs identified at the 5´UTR leader sequence

	**RNAplex**		**RNAup**		**IntaRNA**	
**SARS-CoV-2 leader motif**	**Sites**	**E-value**	**Sites**	**E-value**	**Sites**	**E-value**
UnUnGAUCUnU	16	8.5e-038	14	8.2e-028	19	5.9e-052
AACnAAC	11	6.4e-014	6	2.9e-004	8	4.8e-005
AUACCUUCCCA	4	1.4e-002	3	1.8e-001	6	1.0e-003

**MiRNAs recognizing the UnUnGAUCUnU leader motif**					
**IntaRNA**		**RNAup**		**RNAplex**	
**miRNA**	**p-value**	**miRNA**	**p-value**	**miRNA**	**p-value**
hsa-miR-10397-3p	5.54e-6	-	-	-	-
hsa-miR-1197	5.11e-7	-	-	-	-
hsa-miR-1303	5.11e-7	-	-	-	-
hsa-miR-3141	5.11e-7	hsa-miR-3141	4.54e-7	hsa-miR-3141	2.49e-7
hsa-miR-3144-5p	5.11e-7	-	-	hsa-miR-3144-5p	2.49e-7
hsa-miR-3605-5p	5.11e-7	hsa-miR-3605-5p	4.54e-7	hsa-miR-3605-5p	2.49e-7
hsa-miR-3686	5.54e-6			hsa-miR-3686	6.12e-6
hsa-miR-4433a-3p	5.54e-6	hsa-miR-4433a-3p	7.48e-6	-	-
hsa-miR-4527	5.54e-6	-	-	-	-
hsa-miR-4531	5.11e-7	-	-	-	-
**hsa-miR-4644**	**5.11e-7**	-	-	**hsa-miR-4644**	**2.49e-7**
hsa-miR-4652-5p	5.11e-7	-	-	hsa-miR-4652-5p	2.49e-7
hsa-miR-5004-5p	5.11e-7	-	-	hsa-miR-5004-5p	2.49e-7
**hsa-miR-5192**	**5.11e-7**	**hsa-miR-5192**	**4.54e-7**	**hsa-miR-5192**	**6.12e-6**
**hsa-miR-5572**	**5.11e-7**	-	-	-	-
**hsa-miR-6716-5p**	**5.11e-7**	-	-	-	-
hsa-miR-6857-5p	5.11e-7	-	-	hsa-miR-6857-5p	2.49e-7
**hsa-miR-6871-5p**	**5.54e-6**	**hsa-miR-6871-5p**	**7.48e-6**	**hsa-miR-6871-5p**	**6.12e-6**
-	-	**hsa-miR-377-5p**	**7.48e-6**	**hsa-miR-377-5p**	**6.12e-6**
hsa-miR-7154-3p	5.11e-7	hsa-miR-7154-3p	4.54e-7	hsa-miR-7154-3p	2.49e-7
-	-	hsa-miR-9900	4.54e-7	hsa-miR-9900	2.49e-7
-	-	-	-	hsa-miR-4768-3p	6.12e-6
-	-	hsa-miR-6133	7.48e-6	-	-
-	-	hsa-miR-6529-5p	4.54e-7	-	-
-	-	hsa-miR-6780a-5p	7.48e-6	-	-
-	-	**hsa-let-7c-5p**	**7.48e-6**	-	-
hsa-miR-3123	5.11e-7	hsa-miR-3123	7.48e-6	hsa-miR-3123	2.49e-7
-	-	-	-	hsa-miR-1283	6.12e-6
-	-	**hsa-miR-30c-1-3p**	**7.48e-6**	**hsa-miR-30c-1-3p**	**2.49e-7**
hsa-miR-3976	5.11e-7	hsa-miR-3976	4.54e-7		

**MiRNAs recognizing the AACnAAC leader motif**					
**IntaRNA**		**RNAup**		**RNAplex**	
**miRNA**	**p-value**	**miRNA**	**miRNA**	**p-value**	**miRNA**
-	-	-	-	**hsa-let-7c-5p**	**1.77e-5**
hsa-miR-219a-1-3p	1.16e-5	-	-	hsa-miR-219a-1-3p	1.77e-5
hsa-miR-296-3p	1.16e-5	-	-	hsa-miR-296-3p	1.77e-5
**hsa-miR-377-5p**	**1.16e-5**	-	-	-	-
**hsa-miR-4464**	**1.72e-5**	**hsa-miR-4464**	**1.85e-6**	**hsa-miR-4464**	**2.16e-5**
hsa-miR-449a	1.46e-4	hsa-miR-449a	3.59e-6	hsa-miR-449a	7.33e-6
hsa-miR-495-5p	1.16e-5	-	-	-	-
hsa-miR-5087	7.71e-5	-	-	hsa-miR-5087	6.95e-5
**hsa-miR-6871-5p**	**1.16e-5**	-	-	-	-
-	-	**hsa-miR-6752-5p**	**3.59e-6**	**hsa-miR-6752-5p**	**7.33e-6**
-	-	hsa-miR-6506-3p	1.85e-6	-	-
-	-	-	-	**hsa-miR-5192**	**1.77e-5**
-	-	-	-	hsa-miR-6751-5p	1.77e-5
-	-	-	-	hsa-miR-6823-5p	7.33e-6
**hsa-miR-30c-1-3p**	**1.16e-5**	-	-	**hsa-miR-30c-1-3p**	**1.77e-5**
**MiRNAs recognizing the AUACCUUCCCA leader motif**					
**IntaRNA**		**RNAup**		**RNAplex**	
**miRNA**	**p-value**	**miRNA**	**miRNA**	**p-value**	**miRNA**
hsa-miR-204-3p	2.24e-5	hsa-miR-204-3p	1.09e-6	hsa-miR-204-3p	7.37e-7
-	-	hsa-miR-6825-5p	1.09e-6	hsa-miR-6825-5p	7.37e-7
hsa-miR-7155-5p	2.24e-5	-	-	hsa-miR-7155-5p	7.37e-7
hsa-miR-4314	2.24e-5	-	-	-	-
hsa-miR-7851-3p	2.24e-5	-	-	-	-
hsa-miR-890	2.24e-5	-	-	-	-
hsa-miR-1343-3p	2.24e-5	hsa-miR-1343-3p	1.09e-6	-	-
-	-	**hsa-miR-6716-5p**	**1.09e-6**	-	-
-	-	-	-	hsa-miR-30c-1-3p	7.37e-7
hsa-miR-3661	2.24e-5	hsa-miR-3661	1.09e-6	hsa-miR-3661	7.37e-7
**hsa-miR-6752-5p**	**2.24e-5**	-	-	**hsa-miR-6752-5p**	**7.37e-7**
**In bold, miRNAs recognizing more than one motif**					

Table S9. Role of miRNA candidates against Spike transcript and the 3´UTR of SARS-CoV-2

**miRNAs-Spike**	**Reported function**	**Ref.**
hsa-miR-548an	Inhibitor of host antiviral response	[1]
hsa-miR-939-3p	Pro-inflammatory role/extracellular vescicles	[2]
hsa-miR-3145-5p	Inhibitor of H1N1-related protein PB1	[3]
hsa-miR-563	Promoter of cell proliferation by targeting LIN28B	
hsa-miR-3155a	cardiovascular disease biomarker	[4]
hsa-miR-648	Inhibitor of PAH	[5]
hsa-miR-23a-3p	Implicated in the progression of PAH	[6]
hsa-miR-660-3p	Downregulated in lung cancer	[7]
hsa-miR-4678	Reumatic arthritic disease positive biomarker	[8]
hsa-miR-135a-5p	Protects from PAH	[9]
hsa-miR-324-3p	Upregulated at end-stage osteoarthritis	[10]
hsa-miR-708-5p	Potential diagnostic marker of lung cancer	[11]
hsa-miR-627-5p	Inhibits osteosarcoma cell proliferation/downregulated in PAH	[12]
hsa-miR-196a-3p	Promoter of PAH	[13]
hsa-miR-604	Immuno-marker of HBV chronic infection in Korean patients	[14]
hsa-miR-219b-5p	Involved in PAH	[15]
hsa-miR-581	Promotes HBV antigen expression by targeting DICER and EDEM1	[16]
hsa-miR-4500	Inhibitor of PAH	[17]
hsa-miR-19a-3p	Marker of PAH due to congenital heart disease	[18]
hsa-miR-3085-5p	Human osteoarthritis marker and promoter	[19]
hsa-miR-320c	Promoter of PAH	[20]
hsa-miR-661	Promoter of NSCLC	[21]
hsa-miR-7515	Inhibitor of cell proliferation and growth of lung cancer	[22]
hsa-miR-200b-3p	Protects against epithelial-to-mesenchimal transition bronchial cells	[23]
hsa-miR-320d	Marker in inhaled treatment of COPD	[24]
hsa-miR-335-5p	Inhibitor of cell proliferation of NSCLC	[25]
hsa-miR-920	Altered expression in patients with arthritis	
hsa-miR-378a-5p	Affects lipid metabolism and storage	[26]
hsa-miR-555	Anti-viral role	[27]
hsa-miR-425-5p	Controls lipogenesis and lipolysis in adipocytes	[28]
hsa-miR-1287-3p	Inhibits cancer growth	[29]
hsa-miR-7977	Serum marker of lung adenocarcinoma	[30]
hsa-miR-664a-5p	Promotes obstructive pulmonary disease	[31]
hsa-miR-320b	Marker, low levels indicates reduced survival of COPD patients	[32]
**miRNAs-3´UTR**	**Reported function**	**ref**
hsa-miR-1183	Rheumatic heart disease diagnostic biomarker	[8]
hsa-miR-4710	Endothelial cell proliferation	[33]
hsa-miR-183-5p	Immunity as miRNA cluster	[34]
hsa-miR-22-5p	Promotes T_H17 related pulmonary emphysema	[35]
hsa-miR-6835-5p	Promoter of LPS-induced endothelial inflammation	[36]
hsa-miR-6867-3p	Platelets thrombocytopenia	[37]
hsa-miR-1304-3p	Inhibits NSCLC growth [in vitro]	[38]

hsa-miR-130b-5p	NSCLC oncomiRNA	
hsa-miR-6732-5p	Biomarker of nasopharyngeal carcinoma	[39]
hsa-miR-92a-2-5p	Suppresses anti-viral innate immunity/pulmonary inflammation	[40, 41]

PAH: pulmonary arterial hypertension; NSCLC: Nonsmall-cell lung carcinoma; COPD: Chronic obstructive pulmonary disease; HBV: Hepatitis B virus

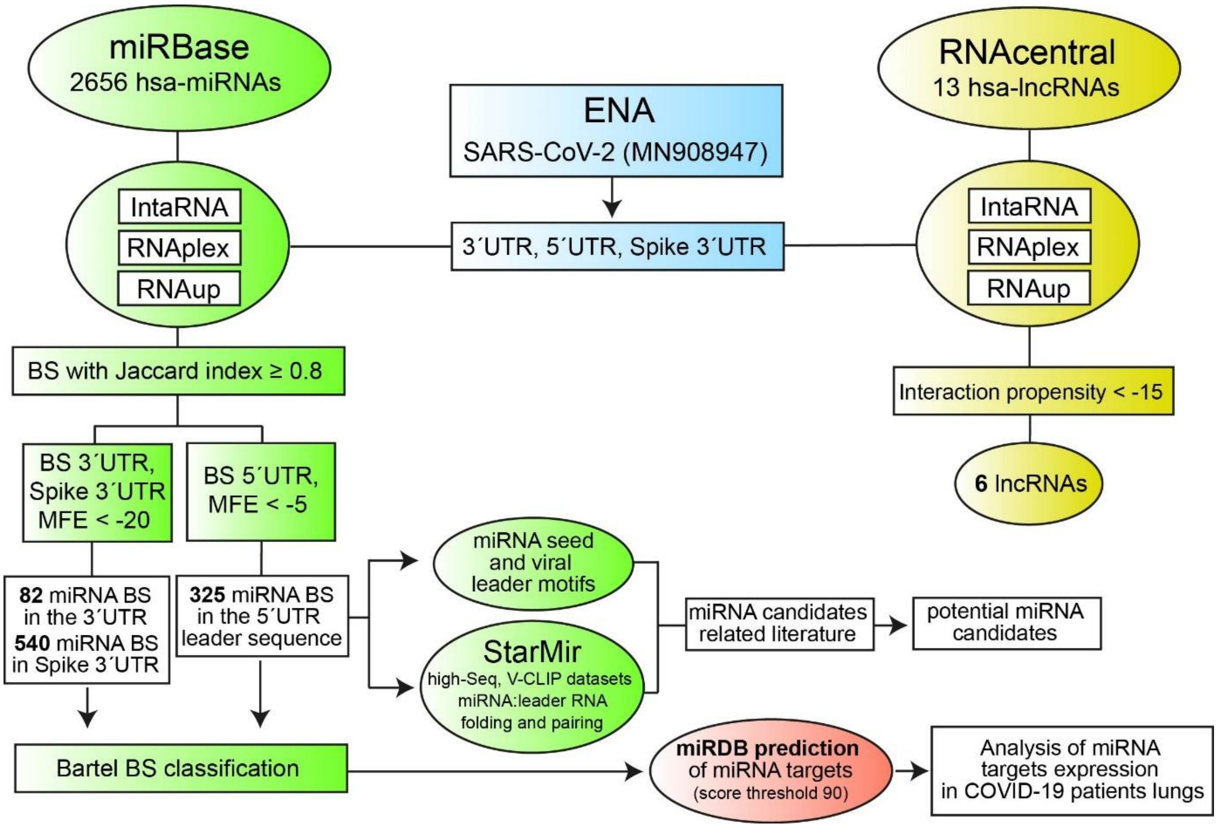

Figure S1. Schematic workflow for the entire study including the parameters/threshold used at each step.

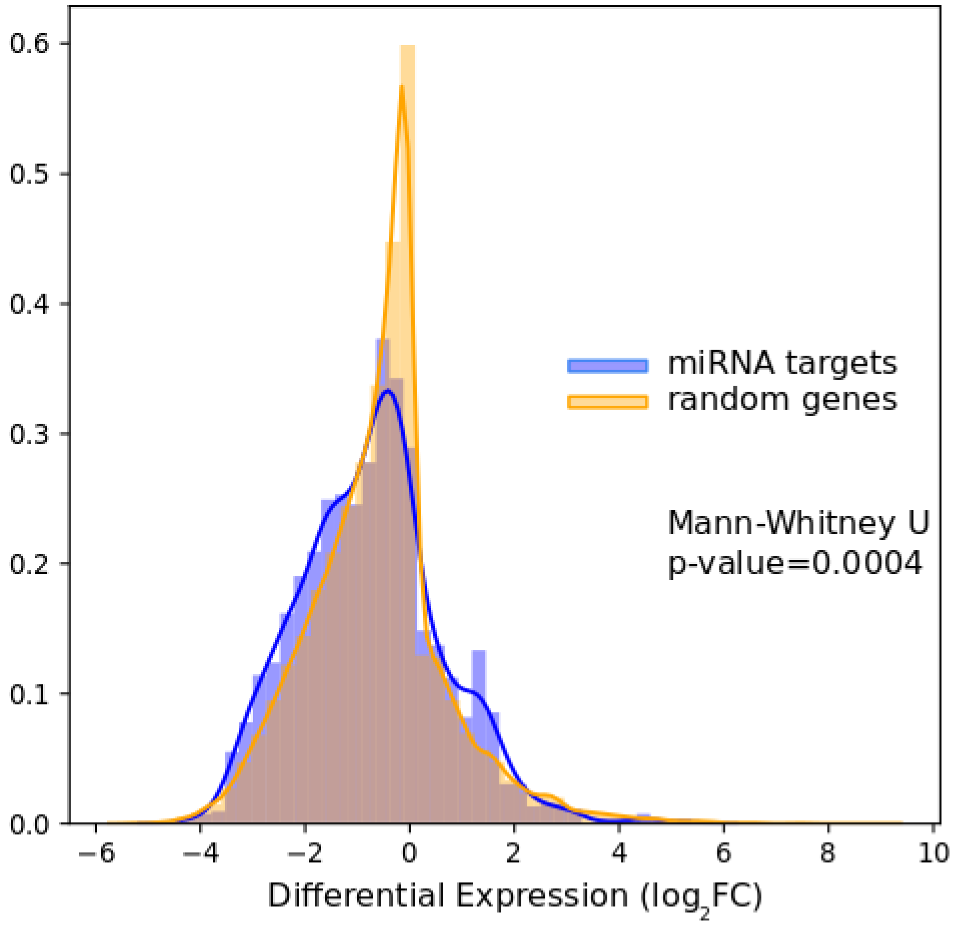

Figure S2. Distribution of the differential expression values (log$_2$ of the expression Fold Change, FC] of genes targeted by the miRNAs targeting the leader sequence of the SARS-CoV-2 virus (blue] and of an equal number of random genes (orange]. Expression values have been collected from Blanco-Melo D. et al. (42]. The two distributions are significantly different (Mann-Whitney U p-value < 0.01].

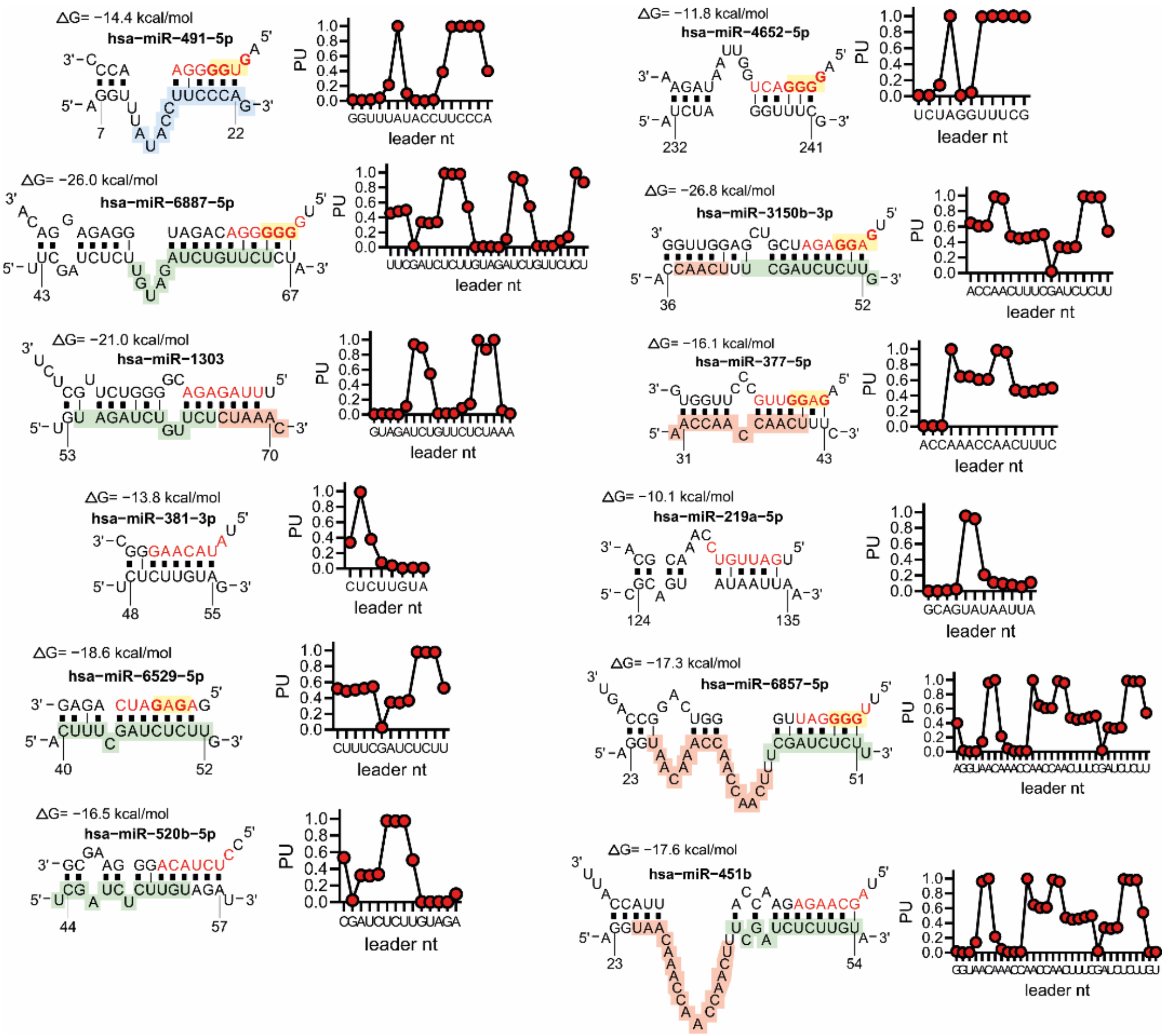

Figure S3. GGG motif and leader motifs from selected miRNA candidates interacting with the leader sequence of SARS-CoV-2. miRNAs reported in Table 1 and known regulators of pulmonary arterial hypertension, atherosclerosis, inflammation, and other diseases. In yellow the GGG motif. In green, red, and light blue the identified leader motifs. Total free energy is represented as ΔG and expressed in kcal/mol. PU: probability of a nucleotide to be unpaired; nt: nucleotide.

Supplementary References

1. Li Y, Xie J, Xu X, Wang J, Ao F, Wan Y, et al. MicroRNA-548 down-regulates host antiviral response via direct targeting of IFN-λ1. Protein Cell. 2013;4(2):130-41.
2. McDonald MK, Ramanathan S, Touati A, Zhou Y, Thanawala RU, Alexander GM, et al. Regulation of proinflammatory genes by the circulating microRNA hsa-miR-939. Scientific Reports. 2016;6(1):30976.
3. Khongnomnan K, Makkoch J, Poomipak W, Poovorawan Y, Payungporn S. Human miR-3145 inhibits influenza A viruses replication by targeting and silencing viral PB1 gene. Exp Biol Med (Maywood). 2015;240(12):1630-9.
4. Ultimo S, Zauli G, Martelli AM, Vitale M, McCubrey JA, Capitani S, et al. Cardiovascular disease-related miRNAs expression: potential role as biomarkers and effects of training exercise. Oncotarget. 2018;9(24):17238-54.
5. Li C, Gonsalves CS, Eiymo Mwa Mpollo M-S, Malik P, Tahara SM, Kalra VK. MicroRNA 648 Targets ET-1 mRNA and Is Cotranscriptionally Regulated with MICAL3 by PAX5. Molecular and Cellular Biology. 2015;35(3):514-28.
6. Sarrión I, Juan G, Ramón M, Cortijo J, Mata M. Role of mir23a and PGC-1 alpha in pulmonary hypertension. European Respiratory Journal. 2013;42(Suppl 57):P5153.
7. Fortunato O, Boeri M, Moro M, Verri C, Mensah M, Conte D, et al. Mir-660 is downregulated in lung cancer patients and its replacement inhibits lung tumorigenesis by targeting MDM2-p53 interaction. Cell Death & Disease. 2014;5(12):e1564-e.
8. Li N, Lian J, Zhao S, Zheng D, Yang X, Huang X, et al. Detection of Differentially Expressed MicroRNAs in Rheumatic Heart Disease: miR-1183 and miR-1299 as Potential Diagnostic Biomarkers. BioMed research international. 2015;2015:524519.

9. Yan X, Li W, Yang L, Dong W, Chen W, Mao Y, et al. MiR-135a Protects Vascular Endothelial Cells Against Ventilator-Induced Lung Injury by Inhibiting PHLPP2 to Activate PI3K/Akt Pathway. Cellular Physiology and Biochemistry. 2018;48(3]:1245-58.
10. Woods S, Barter MJ, Elliott HR, McGillivray CM, Birch MA, Clark IM, et al. miR-324-5p is up regulated in end-stage osteoarthritis and regulates Indian Hedgehog signalling by differing mechanisms in human and mouse. Matrix Biology. 2019;77:87-100.
11. Jang JS, Jeon HS, Sun Z, Aubry MC, Tang H, Park CH, et al. Increased miR-708 expression in NSCLC and its association with poor survival in lung adenocarcinoma from never smokers. Clinical cancer research : an official journal of the American Association for Cancer Research. 2012;18(13]:3658-67.
12. Kheyfets VO, Sucharov CC, Truong U, Dunning J, Hunter K, Ivy D, et al. Circulating miRNAs in Pediatric Pulmonary Hypertension Show Promise as Biomarkers of Vascular Function. Oxid Med Cell Longev. 2017;2017:4957147-.
13. Guerriero I, D'Angelo D, Pallante P, Santos M, Scrima M, Malanga D, et al. Analysis of miRNA profiles identified miR-196a as a crucial mediator of aberrant PI3K/AKT signaling in lung cancer cells. Oncotarget. 2017;8(12]:19172-91.
14. Sarkar N, Chakravarty R. Hepatitis B Virus Infection, MicroRNAs and Liver Disease. Int J Mol Sci. 2015;16(8]:17746-62.
15. Zhou G, Chen T, Raj JU. MicroRNAs in pulmonary arterial hypertension. American journal of respiratory cell and molecular biology. 2015;52(2]:139-51.
16. Wang Y-Q, Ren Y-F, Song Y-J, Xue Y-F, Zhang X-J, Cao S-T, et al. MicroRNA-581 promotes hepatitis B virus surface antigen expression by targeting Dicer and EDEM1. Carcinogenesis. 2014;35(9]:2127-33.
17. Zhang L, Qian J, Qiang Y, Huang H, Wang C, Li D, et al. Down-Regulation of miR-4500 Promoted Non-Small Cell Lung Cancer Growth. Cellular Physiology and Biochemistry. 2014;34(4]:1166-74.
18. Chen W, Li S. Circulating microRNA as a Novel Biomarker for Pulmonary Arterial Hypertension Due to Congenital Heart Disease. Pediatric Cardiology. 2017;38(1]:86-94.
19. Crowe N, Swingler TE, Le LTT, Barter MJ, Wheeler G, Pais H, et al. Detecting new microRNAs in human osteoarthritic chondrocytes identifies miR-3085 as a human, chondrocyte-selective, microRNA. Osteoarthritis Cartilage. 2016;24(3]:534-43.
20. Matamala N, Otero Á, Vázquez I, Retana D, Muñoz-Callejas A, Fernández-González T, et al. MiR-320c regulates SERPINA1 expression and is associated with pulmonary disease in alpha-1-antitrypsin deficiency. European Respiratory Journal. 2016;48(suppl 60]:PA3406.
21. Liu F, Cai Y, Rong X, Chen J, Zheng D, Chen L, et al. MiR-661 promotes tumor invasion and metastasis by directly inhibiting RB1 in non small cell lung cancer. Molecular Cancer. 2017;16(1]:122.
22. Lee JM, Yoo JK, Yoo H, Jung HY, Lee DR, Jeong HC, et al. The novel miR-7515 decreases the proliferation and migration of human lung cancer cells by targeting c-Met. Molecular cancer research : MCR. 2013;11(1]:43-53.
23. Ladak SS, Roebuck E, Powell J, Fisher AJ, Ward C, Ali S. The Role of miR-200b-3p in Modulating TGF-β1–induced Injury in Human Bronchial Epithelial Cells. Transplantation. 2019;103(11].
24. Faiz A, Steiling K, Roffel MP, Postma DS, Spira A, Lenburg ME, et al. Effect of long-term corticosteroid treatment on microRNA and gene-expression profiles in Chronic Obstructive Pulmonary Disease. European Respiratory Journal. 2019:1801202.
25. Tang H, Zhu J, Du W, Liu S, Zeng Y, Ding Z, et al. CPNE1 is a target of miR-335-5p and plays an important role in the pathogenesis of non-small cell lung cancer. Journal of Experimental & Clinical Cancer Research. 2018;37(1]:131.
26. Krist B, Florczyk U, Pietraszek-Gremplewicz K, Józkowicz A, Dulak J. The Role of miR-378a in Metabolism, Angiogenesis, and Muscle Biology. International Journal of Endocrinology. 2015;2015:281756.
27. Shim B-S, Wu W, Kyriakis CS, Bakre A, Jorquera PA, Perwitasari O, et al. MicroRNA-555 has potent antiviral properties against poliovirus. Journal of General Virology. 2016;97(3]:659-68.
28. Qi R, Wang J, Wang Q, Qiu X, Yang F, Liu Z, et al. MicroRNA-425 controls lipogenesis and lipolysis in adipocytes. Biochimica et Biophysica Acta (BBA] - Molecular and Cell Biology of Lipids. 2019;1864(5]:744-55.
29. Schwarzenbacher D, Klec C, Pasculli B, Cerk S, Rinner B, Karbiener M, et al. MiR-1287-5p inhibits triple negative breast cancer growth by interaction with phosphoinositide 3-kinase CB, thereby sensitizing cells for PI3Kinase inhibitors. Breast Cancer Research. 2019;21(1]:20.
30. Chen L, Cao P, Huang C, Wu Q, Chen S, Chen F. Serum exosomal miR-7977 as a novel biomarker for lung adenocarcinoma. Journal of cellular biochemistry. 2020;121.
31. Zhong S, Chen C, Liu N, Yang L, Hu Z, Duan P, et al. Overexpression Of hsa-miR-664a-3p Is Associated With Cigarette Smoke-Induced Chronic Obstructive Pulmonary Disease Via Targeting FHL1. Int J Chron Obstruct Pulmon Dis. 2019;14:2319-29.
32. Keller A, Ludwig N, Fehlmann T, Kahraman M, Backes C, Kern F, et al. Low miR-150-5p and miR-320b Expression Predicts Reduced Survival of COPD Patients. Cells. 2019;8(10]:1162.
33. Vidal-Gómez X, Pérez-Cremades D, Mompeón A, Dantas AP, Novella S, Hermenegildo C. MicroRNA as Crucial Regulators of Gene Expression in Estradiol-Treated Human Endothelial Cells. Cellular physiology and biochemistry : international journal of experimental cellular physiology, biochemistry, and pharmacology. 2018;45(5]:1878-92.
34. Ichiyama K, Dong C. The role of miR-183 cluster in immunity. Cancer letters. 2019;443:108-14.
35. Lu W, You R, Yuan X, Yang T, Samuel ELG, Marcano DC, et al. The microRNA miR-22 inhibits the histone deacetylase HDAC4 to promote T(H]17 cell-dependent emphysema. Nat Immunol. 2015;16(11]:1185-94.

36. Liu J, Li G, Chen C, Chen D, Zhou Q. MiR-6835 promoted LPS-induced inflammation of HUVECs associated with the interaction between TLR-4 and AdipoR1 in lipid rafts. PLoS One. 2017;12(11]:e0188604.
37. Deng G, Yu S, He Y, Sun T, Liang W, Yu L, et al. MicroRNA profiling of platelets from immune thrombocytopenia and target gene prediction. Molecular medicine reports. 2017;16(3]:2835-43.
38. Li C-G, Pu M-F, Li C-Z, Gao M, Liu M-X, Yu C-Z, et al. MicroRNA-1304 suppresses human non-small cell lung cancer cell growth in vitro by targeting heme oxygenase-1. Acta Pharmacol Sin. 2017;38(1]:110-9.
39. Li K, Zhu X, Li L, Ning R, Liang Z, Zeng F, et al. Identification of non-invasive biomarkers for predicting the radiosensitivity of nasopharyngeal carcinoma from serum microRNAs. Scientific Reports. 2020;10(1]:5161.
40. Sheng Y, Wang Y, Lu W, Zhou Y, Dong G, Ge X, et al. MicroRNA-92a inhibits macrophage antiviral response by targeting retinoic acid inducible gene-I. Microbiology and immunology. 2018;62(9]:585-93.
41. Fu L, Zhu P, Qi S, Li C, Zhao K. MicroRNA-92a antagonism attenuates lipopolysaccharide (LPS]-induced pulmonary inflammation and injury in mice through suppressing the PTEN/AKT/NF-κB signaling pathway. Biomedicine & Pharmacotherapy. 2018;107:703-11.
42. Blanco-Melo D, Nilsson-Payant BE, Liu WC, Uhl S, Hoagland D, Møller R, et al. Imbalanced Host Response to SARS-CoV-2 Drives Development of COVID-19. Cell. 2020;181(5]:1036-45.e9.
